# Deciphering the Mechanical Network of Chronic Atrophic Gastritis: A Urinary Time-Dependent Metabonomics-Based Network Pharmacology Study

**DOI:** 10.3389/fphys.2019.01004

**Published:** 2019-08-06

**Authors:** YueTao Liu, WenQian Xu, XueMei Qin

**Affiliations:** Modern Research Center for Traditional Chinese Medicine of Shanxi University, Taiyuan, China

**Keywords:** chronic atrophic gastritis, biomarkers, metabonomics, ANOVA-simultaneous component analysis, network pharmacology

## Abstract

Chronic atrophic gastritis (CAG) is one of the most important pre-cancerous states with a high prevalence. Deciphering its mechanical network is of significant importance for its diagnosis and treatment. The time-series factor associated with CAG progression specially needs to be considered together with its biological condition. In the present work, ^1^H NMR-based dynamic metabonomics was firstly performed to analyze the urinary metabolic features of CAG coupled with ANOVA-simultaneous component analysis (ASCA). As results, 4 (alanine, lipids, creatine, and dimethylglycine), 2 (α-ketoglutarate and alanine) and 5 (succinate, α-ketoglutarate, alanine, hippurate, and allantoin) urine metabolites were finally selected as the candidate biomarkers related to phenotype, time, and their interaction, respectively. Mechanistically, the network pharmacology analysis further revealed these metabolites were involved into mitochondrial function, oxidation reduction, cofactor binding, generation of precursor metabolites and energy, nucleotide binging, coenzyme metabolic process, cofactor metabolic process, cellular respiration, and tricarboxylic acid cycle. Especially, mitochondria were the most important targeted organelle referred 30 targeted proteins. The present work provided a novel network pharmacology approach for elucidating the mechanisms underlying the pathogenesis of CAG based on urinary time dependent metabonomics.

## Introduction

Chronic atrophic gastritis (CAG) is an inflammatory disease of the stomach from various etiologies (Ferlay et al., 2002, 2005; Weck and Brenner, 2006). Typical symptoms, when present, include epigastric pain, fullness, belching, anorexia, and other non-specific symptoms. CAG can lead to mucosal atrophy, intestinal metaplasia (IM), and gastric intraepithelial dysplasia (GED), also known as intraepithelial neoplasia, which is defined as the precancerous stage of gastric carcinoma (Asaka et al., 2001). Global cancer statistics for 2012 estimated that there were 951,600 new cases of stomach cancer worldwide (Torre et al., 2015). The transition from chronic gastritis to gastric cancer is a typical disease model of uncontrolled inflammation leading to malignant transformation. Preclinical research thus seems essential to probe the mechanisms related to CAG biology. Modern research in the exploration of pathogenesis is moving from classical methodologies to the characterization of the metabolome variation induced by CAG (Chan et al., 2014).

In recent years, studies on the metabolic disturbances of CAG, have been reported with the aims to interpret its biochemical process. A lot of potential CAG biomarkers in bio-fluid samples were identified relating to energy metabolism, inflammation, immune dysfunction, and oxidative injury, which were matched with the relevant pathological changes in the formation of CAG (Cui et al., 2017; Liu et al., 2017). However, metabonomics, a relatively new “omics” technique that attempts to profile all low-molecular weight metabolites, are often used in longitudinal studies such as monitoring disease progression, tracking nutritional interventions, or observing drug toxicity. So other factors (such as the time-series for their process) together with the biological condition of interest need to consider during data analysis. Special designed statistical approach based on ANOVA-simultaneous component analysis (ASCA) has been developed to deal with the multifactor issues as well as time-series studies (Smilde et al., 2005; Zwanenburg et al., 2011). It has been successfully applied to analyze metabonomic data. Gao et al. (2010) used ASCA to investigate the effects of Aβ_25__–__35_ injection, time, and their interaction on the hippocampus and serum metabolome (Du et al., 2017). ASCA was also used to examine different sources of the data related to each experimental factor (defined as time, diet, and individual) to assess dietary influence on type 2 diabetes development, where showed a significant contribution of the time-diet interaction factor (Ly-Verdú et al., 2015). Meanwhile, network pharmacology is a bioinformatics strategy to map the disease mechanical networks from the biological level (Zheng et al., 2018). Increasing evidences also showed its potential on illustrating the molecular mechanisms of the complex disease.

In this study, two factors and their interaction, CAG (phonotype) and time, were included to screen the metabonomic biomarkers associated with CAG in a rat model over time. Thus, ASCA was selected, since it can not only include underlying multiple factors and their interaction, but also facilitate to interpret the various effects of different factors. We constructed CAG rat models and examined the urinary time-dependent metabonomic analysis using NMR. Then, network pharmacology was used to decipher the mechanical network of CAG based on the obtained potential metabolic biomarkers related to CAG, time, and their interaction.

## Materials and Methods

### Reagents and Materials

Deuterium oxide with 0.05% 3-trimethylsilyl-(2, 2, 3, 3-2H4)-1-propionate (TSP) (D_2_O, 99.9%) was purchased from Sigma-Aldrich (St. Louis, MI, United States). Sodium deoxycholate was provided by Beijing Aoboxing Bio-tech, Co., Ltd., (Beijing, China). The assay kit for pepsin activity (PA) was purchased from Nanjing Jiancheng Bioengineering Institute (Nanjing, China). Ultrapure water (18.2 MΩ) was prepared with a Milli-Q water purification system (Millipore, Molsheim, France). All other used chemicals were of analytical grade.

### Animal Treatment

All procedures for animal treatment were in accordance with the National Guidelines for Experimental Animal Welfare (MOST, China, 2006) at the Center for Animal Experiments, which had full accreditation from Animal Ethics Committee of Shanxi University and were exerted to minimize animal suffering and the number of animals necessary for the attainment of reliable data. SPF-grade male Sprague-Dawley (SD) rats (body weight, 180 ± 20 g), were obtained from Vital River Laboratory Animal Technology Co., Ltd., (Beijing, China). They were maintained at a constant humidity (ca. 60%) and temperature (ca. 23°C) with a light/dark cycle of 12 h.

After 1 week of adaptation, the rats were randomly separated into 2 groups according to the body weights (*n* = 6), control group and CAG group. The replication of CAG rat model was performed according to Zhang’ experimental method with some modifications (Zhang et al., 2013). From the 1st day, those rats in model group were administrated freely with ammonia solution (0.1%) and sodium deoxycholate (20 mmol/L) on alternate days, respectively. Meanwhile, the animals were treated with the hunger disorder method, which rats had free access to normal diet for 2 days, and then fasted for 1 day. The cycles were performed during the whole experimental period of 10 weeks. The control group had free access to normal chow and water. Body weights were measured every 6 day in 1st month and every 3 day in the followed experimental period.

### Sample Collection

Urine samples were collected individually at 0-, 4-, 6-, 8-, and 10-week in metabolic cages for 24 h urinary (containing 0.05% sodium azide) collection after a 12 h fast. Whole urine samples were centrifuged for 10 min at 3,333 *g*. And then the supernatant was carefully collected in fresh polypropylene tubes, stored at −80°C for further analysis. After the last body weight determination at the 10th week, rats were anesthetized with 10% urethane. Blood samples were collected, and centrifuged at 2,500 *g* for 15 min at 4°C. The resultant plasma samples were stored at −80°C for PA analysis. The gastric tissues were immediately removed and washed with physiological saline. One part of gastric tissues was cut and put into a tube containing 10% formaldehyde solution for the histopathology analysis.

### Biochemistry Assays and Histological Examination

Biochemical index of gastric PA was measured according to the instruction of enzymatic kit. Gastric tissues were fixed with 10% formaldehyde solution for 48 h, embedded in paraffin, 5 mm sectioned, and stained with hematoxylin-eosin (HE). Images were obtained and studied under light microscopy (Olympus BX53, Tokyo, Japan).

### Urinary Sample Preparation and NMR Analysis

Five hundred microliter of urine was mixed with 200 μL phosphate buffer (0.2 M Na_2_HPO_4_/NaH_2_PO_4_, pH 7.4) containing D_2_O for the purpose of field lock and TSP as a chemical shift reference. The whole mixtures were eddied for 30 s, and centrifuged at 14,800 *g* for 15 min (4°C). Finally, 550 μL of sample supernatant was placed in a 5 mm NMR tube for NMR analysis.

The one dimensional (1D) NMR spectra and two-dimensional (2D) NMR spectra were recorded at 298K on a Bruker 600 MHz AVANCE III NMR spectrometer (Bruker BioSpin, Bremen, Germany) equipped with a Bruker 5 mm PA BBO probe operated at 600.13 MHz ^1^H frequency. Samples were analyzed using nuclear over hauser effect spectroscopy (NOESY, RD-90°-t1-90°-tm-90°-acquire) NMR spectra with water suppression. Each ^1^H NMR spectrum of urine consisted of 64 scans requiring a 2.654 s acquisition time with the following parameters: spectral width of 12345.7 Hz, spectral size of 65536 points, and a relaxation delay (RD) of 1.0 s.

For spectral assignment purposes, two-dimensional (2D) NMR spectra including ^1^H-^1^H correlation spectroscopy (COSY), ^1^H-^13^C heteronuclear single-quantum correlation spectroscopy (HSQC) were recorded. 2D ^1^H-^1^H COSY spectra were analyzed using the noesygpprqf pulse sequence for urine samples and following parameters: 1.5 s RD and 6602.1 Hz spectral width in F2 and 6601.5 Hz in F1. 2D ^1^H-^13^C HSQC spectra were analyzed using the hsqcetgpsisp pulse sequence for urine samples and following parameters: 1.2 s RD and 6602.1 Hz spectral width in F2 and 36220.3 Hz in F1.

Metabolite peaks were interpreted with available biochemical databases, such as HMDB^1^ and KEGG^2^. Further validations were achieved by extensive analysis of 2D NMR spectra (COSY and HSQC), and the cross peaks in HSQC were input in COLMAR ^13^C-^1^H Query server^3^. The cutoff value for ^1^H and ^13^C were set as 0.06 and 0.6 ppm, respectively. These results were manually checked by interactive user interface using the “Show Me” button, as well as the parameter of matching ratio and uniqueness.

### Data Processing and MetaboAnalyst Analysis

The collective 1D NMR spectra were corrected for phase and baseline distortions using MestReNova (version 8.0.1, Mestrelab Research, Santiago de Compostella, Spain). The 1D NMR spectra of urine were referenced to the chemical shift of TSP (δ 0.00 ppm), respectively. The regions δ 0.50–9.00 ppm was reduced into integral bins of equal width (0.001 ppm). The region of δ 4.68–5.19 ppm was excluded from the analysis to eliminate the effect of imperfect water saturation.

And then the generated data was modified to be the accepted data formats described in the “Time-series/Two-factor Design” module of MetaboAnalyst analysis^4^ (Xia and Wishart, 2016), which contained multiple types of variation among phenotype (CAG), time, and their interaction.

The dataset was first normalized to constant sum and pareto scaled, and then introduced into ASCA, which was used to investigate the different variations including phenotype, time, and their interaction. Its major advantage was that each sub model can be analyzed separately without being confounded with the other variation sources. SPE (squared prediction error) and Leverage were proposed to evaluate the fitness of the model. SPE was used to test the fitness of a model for the metabolite. Leverage was used to evaluate the importance of the metabolite to the model. Variables with low SPE and higher leverage should have significant contributions to the model and were picked out as influentially affected metabolites.

### Biological Function Analysis of Targeted Proteins Based on Network Pharmacology

To further analyze the important roles of the potential biomarkers, their upstream proteins were firstly data-mined from Metscape of cytoscape, which was an open source software to integrate biomolecular interaction networks with high-through put expression data into a unified conceptual framework based on KEGG (Gao et al., 2010). Meanwhile, the proteins related with CAG were collected to characterize the pathological protein network of CAG based on OMIM^5^ and Gene Cards databases^6^. These collected proteins were all imported to STRING for protein-protein interactions analysis (PPIs) to link their interactive actions. In such a network, a node represented a protein, and the relationship between them is represented by the lines between the nodes.

To analyze the synthetic biological functional annotation information, the screened proteins were corrected to be their official gene symbol, and imported into DAVID database^7^, which could find out the most significant enrichment biological annotation. Homo sapiens were selected as the restricted specie. The molecular function information of the genes was detected based on the Gene Ontology analysis. The charts were imported out as Excel format with a threshold (*p* < 0.05).

### Statistical Analysis

All values were expressed as mean ± SD. A two-tailed unpaired *t*-test by SPSS 16.0 (Chicago, IL, United States) was applied to analyze those significant differences between two groups including the 0th week and the other individual groups. The value of *p* < 0.05 was considered statistically significant.

## Results

### Weight Change, Clinical Biochemistry, and Histopathology

**FIGURE 1:**
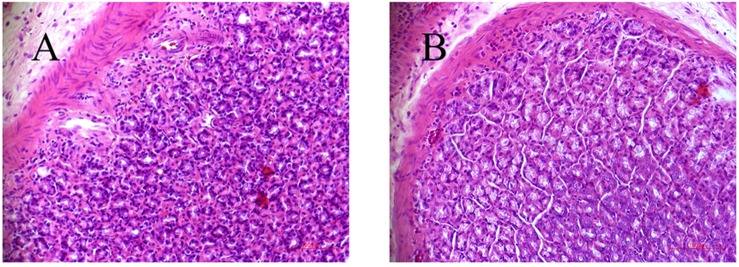
Histological examination of gastric tissues from control **(A)** and CAG **(B)** rats.

As depicted in our previous study (Cui et al., 2017), the weight growth tendency of CAG rats were significantly slower compared with control rats, which was an obvious symptom of CAG. Meanwhile, the level of PA was decreased by 43.8% in CAG group at the 10th week (*p* < 0.01). The gastric mucosa histopathology of model rat was also markedly changed, where glossy gastric mucosa folds were flat or disappeared with pale appearance. Light microscope showed that irregular arrangement and reduction layer of gastric gland in model rats, while increasing thickness of musculoris mucosa, with partly infiltration of eosinophilic cells, inherent glands reduction, and significant atrophy (Figure 1).

### Candidate Biomarkers Screening Based on ASCA Analysis

Thirty-two metabolites were firstly identified based on the available biochemical databases and human metabolome database (HMDB) (Supplementary Table S1). Example of the typical 1D and 2D NMR spectrum was showed in Supplementary Figures S1, S2. 853 variables were finally obtained from urine samples after normalization for the followed ASCA. Supplementary Figure S3 showed the effects before and after normalization, which obtained remarkable improvement in the data structure. Here, ASCA was applied to analyze the urinary dynamic metabonomic data consisting of different variations associated with the animals experiment (phenotype, CAG), time and their interaction (Supplementary Figure S4). Supplementary Figure S5 showed the validation of the ASCA model by a permutation approach (*p* < 0.05), which indicated that the applied model was suited for the following the candidate biomarkers screening. The major patterns based on PC1 of the corresponding sub models were showed in the score scatter plots, which were associated with phenotype factor, time factor, and their interaction, respectively, (Supplementary Figure S6). The results indicated that the abilities of the corresponding sub model were enough to explain the original variables and the model were stable and reliable for the time-dependent metabonomics data.

**FIGURE 2:**
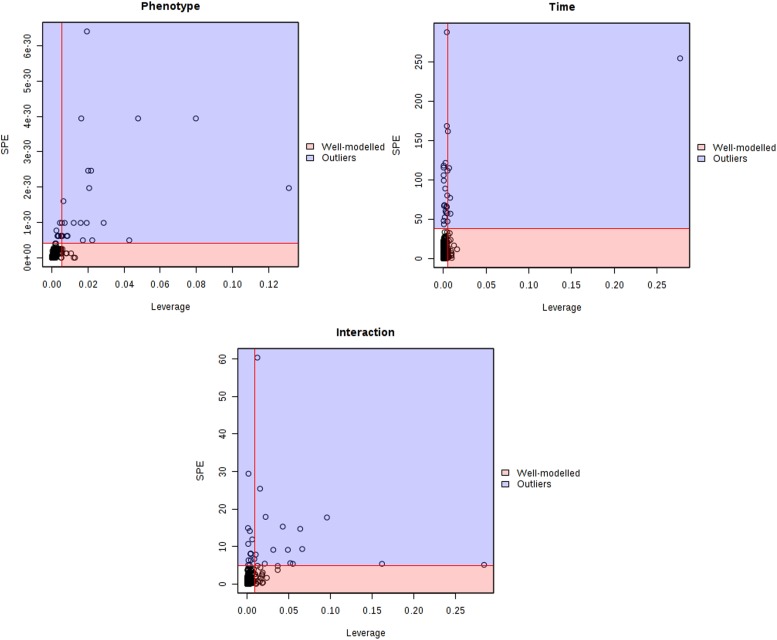
Leverage/SPE scatter plots of the ASCA variables submodels for phenotype, time and their interactions. Metabolites in red region have high loadings that follow the expression patterns of the submodels. Metabolites in blue region have expression patterns that are different from the major patterns.

The major patterns associated with factor A (phenotype), factor B (time variation), and their interaction graphically displayed the relationship between eigenvalues and factors (Figure 2). The significant variables associated with a specific factor were identified based on the leverage/SPE plots. Table 1 showed the identified metabolites well-modeled by the ASCA procedure according to the factors for the phenotype, time and their interaction. A total of 8 metabolites were assigned based on the literature and available biochemical databases, and further evaluated by COLMAR ^13^C-^1^H Query server. As results, 4 (alanine, lipids, creatine, and dimethylglycine), 2 (α-ketoglutarate and alanine) and 5 (succinate, α-ketoglutarate, alanine, hippurate, and allantoin) urine metabolites were finally selected as the candidate biomarkers related to leverage, SPE and their interaction, respectively. It was interesting that alanine was the only one contributed to the two experimental factors and their interactions. The metabolite α-ketoglutarate was contributed to the time-dependent response and the interactions with phenotype. Almost of all the screened metabolites were characterized to be relative with phenotype or the interactions with time factor. So the screened eight metabolites were all recognized as the potential biomarkers related to the progression of CAG based on ASCA.

**TABLE 1:** Details of compounds in the Leverage/SPE scatter plots of the ASCA variables submodules for phenotype (a), time (b), and their interactions (c).

**Phenotype**				**Time**				**Interaction**			
**Metabolite**	**Chemical shifts**	**Leverage**	**SPE**	**Metabolite**	**Chemical shifts**	**Leverage**	**SPE**	**Metabolite**	**Chemical shifts**	**Leverage**	**SPE**
Unknown 1	1.19	0.0160	11.6671	Unknown 6	5.26	0.0127	0	Succinate	2.41(s)	0.0365	4.8047
Alanine	3.76(q)	0.0123	16.5474	α-ketoglutarate	2.44(t), 3.02(t)	0.0105	1.23E-31	α-ketoglutarate	3.02(t), 2.44(t)	0.0365	3.7224
Lipids	0.87(m)	0.0083	23.7446	Alanine	1.46(d)	0.0081	1.23E-31	Unknown 8	4.02	0.0234	1.6266
Unknown 2	4.45	0.0066	2.3062	Unknown 7	1.98	0.0057	2.47E-31	Alanine	1.46(d)	0.0179	0.2502
Unknown 3	2.07	0.0059	13.1421					Unknown 9	3.51	0.0177	2.5917
Creatine	3.92(s)	0.0057	2.2696					Allantoin	5.39(s)	0.0151	0.7031
Unknown 4	3.18	0.0055	9.3417					Unknown 1	1.19	0.0128	0.6678
Unknown 5	2.26	0.0054	29.5758					Unknown 10	3.66	0.0107	0.0295
Dimethylglycine	2.93(s)	0.0053	10.9358					Unknown 11	2.83	0.0106	0.2493
								Unknown 7	1.98	0.0106	1.9994
								Hippurate	3.98(d), 7.56(t), 7.85(d)	0.0095	1.8842
											

### Typical Metabonomics Alteration of the Candidate Biomarkers

**FIGURE 3:**
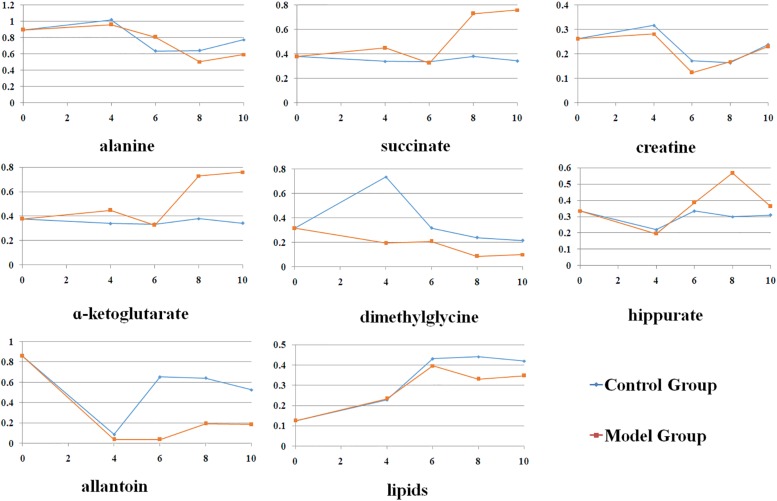
The changes of the endogenous metabolites based on ASCA at different stages.

Figure 3 presented the alterations of the selected metabolites during the whole experiment. It could be observed that most of these metabolites showed slight change from 4th to 10th week in control group, which indicated that the observed changes in metabolic profiles were related to the progression of CAG, but not due to the age of rats. The acute metabolic alteration might be due to the unreliable effect induced by external environment. Compared with normal rats, levels of alanine, succinate, α-ketoglutarate were observed to be maximization of the difference in model rats. At 6th weeks, there were no changes in succinate, α-ketoglutarate levels between them. Meanwhile, creatine was high at this time point and partly restored to control levels during the late stages of CAG. Dimethylglycine was reduced during the progression of CAG, with a drastic reduction seen at 4th week in CAG mice. Compared with the normal rats, allantoin was lower in the CAG rats throughout the experimental period. Additionally, two other metabolites, hippurate and lipids, were changed in CAG rats of 8 weeks. These changes indicated altered gut microbiome might be involved into the development and progression of CAG.

### Biological Function Analysis of Targets Proteins

**FIGURE 4:**
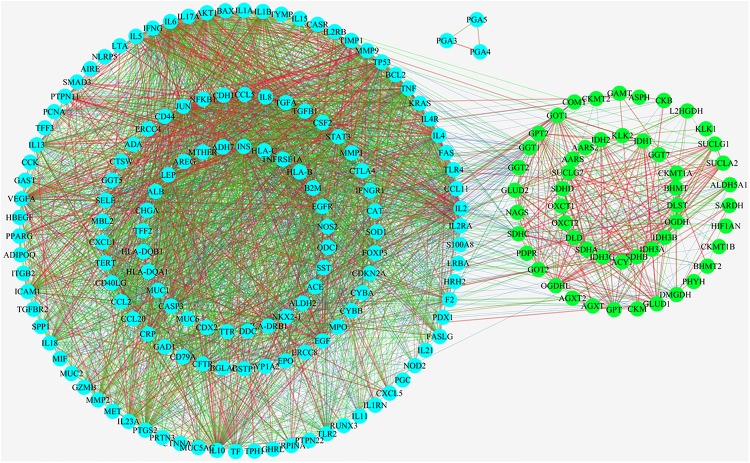
Protein-protein interactions analysis (PPIs) linking their interactive actions of the upstream proteins related to relate to the endogenous metabolites based on ASCA and the collected targeted proteins related to CAG based on OMIM and Genecard databases.

As showed in Figure 4, 57 upstream proteins were collected as the related metabolic targets of CAG based on Metscape (Supplementary Table S2), while 148 targeted proteins related to CAG were found through OMIM and Genecard databases (Supplementary Table S3). Among them, glutamic-pyruvic transaminase (GPT) was both the same one. 98 nodes and 3348 edges were involved and used to construct their PPIs network, while three proteins were not interacted with the others. The thickness and the color of the edge represented the strength of the interaction of these related proteins. Through the network we could intuitively see that there existed the relationships between the upstream targets and the disease targets, which indirectly testified our metabonomics results. Among those 57 upstream proteins, 46 key targeted proteins were screened to analyze their biological functions in the development of CAG, which could linked more targeted proteins related to CAG.

**FIGURE 5:**
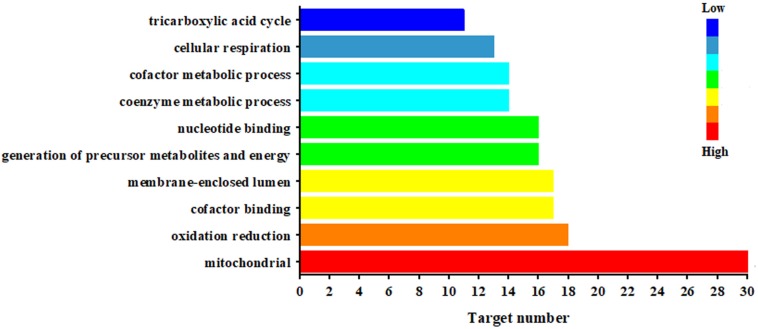
Biological function of the targeted proteins relate to the endogenous metabolites based on ASCA.

Figure 5 illustrated the significantly biological functions and their hits number. The results suggested that the pathogenesis of CAG involved multiple abnormities of biological process, which were related to mitochondrial function, oxidation reduction, cofactor binding, generation of precursor metabolites and energy (17 targets), nucleotide binging, coenzyme metabolic process, cofactor metabolic process, cellular respiration and tricarboxylic acid cycle. Especially, mitochondria were the most important targeted organelle referred 30 targeted proteins.

## Discussion

The common static metabonomics analysis was often used to depict the single cross section of the given pathological state of the disease, without fully considering the dynamic process of their progression. As our previous reported, 18 urinary metabolites, including isoleucine, valine, 3-hydroxybutyrate, acetate, succinate, and α-ketoglutarate, etc., were screened from a single time node (the 10th week) using orthogonal partial least squares discriminate analysis (Cui et al., 2017). These potential biomarkers paved a way for elucidating the underlying mechanisms of CAG. However, these significant variations just depicted a special pathologic state, which did not capture the development of CAG, which is a dynamics process.

Conventionally, metabonomic datasets involved time-series factor are very complex and contain multiple types of variation: the variation originating from differences related to disease that are constant in time, the time-dynamic variation of each individual, or their combinations (Smilde et al., 2010). So it is necessary to separate the original dataset into sub-modules to interpret the systemic effects derived from the biological information. Meanwhile, the observation at different time points and from different replications can be correlated in time-course analysis, which might lead to inaccurate interpretation of the data and prevent achievement of consistent biological conclusions in common static methods.

In the present work based on ASCA, dimethylglycine, succinate, α-ketoglutarate, hippurate and allantoin were all probed in the static and dynamic metabonomics analysis, which were correlative with the factors related to phenotype, time, and their interactions. Another three metabolites only detected through ASCA, alanine, lipids and creatine, were assigned to the phenotype variations. These results suggested that the dynamic metabonomics could discriminate the different variations linked various experimental factors.

Two metabolites (α-ketoglutarate and alanine) were found to be characterized as the time-resolved metabolic biomarkers. Alanine was a new potential biomarker related to the development of CAG, which was not identified from our previous static dynamic metabonomics study (Cui et al., 2017). Alanine has been reported as possible molecular markers related to the human gastric mucosa differentiation toward preneoplastic and neoplastic conditions (Calabrese et al., 2008). The alteration of alanine levels observed in the urine samples of 6- to 10-week-old CAG rats confirmed impaired gastric function. α-Ketoglutarate was an intermediates of tricarboxylic acid (TCA) cycle, could mediate the energy metabolism in the body. Its level was reduction at 4-, 8-, and 10-week in CAG rats, which could be attributed to the dysfunction of TCA cycle in CAG. Analysis of their temporal changes revealed that time has a non-negligible contribution to the total variation. Meanwhile, these two metabolites were also associated with the interactions between phenotype factor and time factor, which further demonstrated that these metabolites underwent time-dependent changes during the progression of CAG.

Four phenotype metabolites, alanine, creatine, dimethylglycine and lipids, were involved into the progression of CAG. Creatine, biosynthesized from arginine, was reported to ascribe its protective effect to energy metabolism and oxidative resistance in gastro biopsy specimens of the antral and corpus mucosa (Gruno et al., 2006). In our study, the decreased creatine at 4-, and 6-week was observed in CAG rat urine samples, indicating that it might be involved into oxidative stress. Dimethylglycine is an anti-stress nutrient with antioxidant properties. Recently, studies have implicated the gastroprotective effect of dimethylglycine could be contributed to its free radical scavenging activity and cytoprotection of gastric mucosa (Bai et al., 2016). The reduction of dimethylglycine during the whole experiment also suggested that the defense of oxidative stress was damaged induced by CAG. Lipids were necessary for cell energy generation and lipid synthesis, which fulfill the cell biological processes. It is reported that inhibition of lipid production was responsible for gastric cancer cell proliferation impairment induced by glycerol uptake reduction (Li et al., 2016). In this study, urine level of lipids was decreased from 6- to 10-week in CAG rats, suggesting the disturbances of lipid play key roles in CAG development.

Five different variations were screened out to depict the interaction between phenotype and time factors based on ASCA. Allantoin, a product of purine metabolism, was an indication of high levels of reactive oxygen species via non-enzymatic means. da Silva et al. (2018) also reported that allantoin could possess gastroprotective activity through anti-inflammatory, anti-oxidative, antisecretory and cytoprotective mechanisms. It was also found to be depressed under CAG condition, indicated that the pathology of inflammatory, oxidative, and secretory participated into the development of CAG. The emerge of hippurate in urine sample indicated that gut flora metabolism was involved into the development of CAG (Diao et al., 2014). Succinate was another intermediates of TCA cycle, suggested that the alterations of energy metabolism played important role under CAG condition. Alaine and α-ketoglutarate were both assigned to the interactive variations. All the result demonstrated that the development of CAG was a dynamic process, where ASCA was a useful tool to illustrate its biochemical process.

In the present work, we have constructed a novel metabonomics-based network pharmacology approach to illustrate the mechanism of CAG linking experimental targets and predicted targets together. ASCA could discriminate the effect of phenotype factor (CAG) from time factor on metabolic alterations, which really characterized the development of disease. Network pharmacology could integrate more comprehensive targets for targets prediction and linking their interactions. 148 proteins were collected to construct the CAG network, which have been reported that played import roles in the pathological development of CAG. PPIs analysis revealed that 3348 links existed the interactions among 198 proteins combining the disease targets and the predicted targets based on the screened metabolites. All these proteins should be recognized as the targets of CAG. GPT, one upstream protein of alanine and α-ketoglutarate, have been reported to associate with upper gastrointestinal bleeding (Moledina and Komba, 2017). Integrating of them could obtain more comprehensive proteins network related to CAG. However, PPIs among predicted targets and experimental targets have not been experimentally proved. Further experimental work can be applied to uncover their relationships.

## Conclusion

In summary, this study aimed to screen the candidate biomarkers associated with the development of CAG integrating of metabonomics, ASCA, and network pharmacology. The systemic effects derived from phenotype, time and their interactions were further exacted based on the application of ASCA, which allowed separating the urinary dynamic metabonomics dataset into related submodels. As results, 4 (alanine, lipids, creatine, and dimethylglycine), 2 (α-ketoglutarate and alanine), and 5 (succinate, α-ketoglutarate, alanine, hippurate, and allantoin) urine metabolites were finally selected as the candidate biomarkers related to phenotype, time and their interaction, respectively. The followed network pharmacology analysis further revealed that the predicted targets were involved into mitochondrial function, oxidation reduction, cofactor binding, generation of precursor metabolites and energy, nucleotide binging, coenzyme metabolic process, cofactor metabolic process, cellular respiration, and tricarboxylic acid cycle. Additional studies are still necessary for further evaluation and validation of the biomarkers identified in the current study.

## Ethics Statement

This study was carried out in accordance with the principles of the Basel Declaration and Recommendations of the National Guidelines for Experimental Animal Welfare (MOST, China, 2006) of the Center for Animal Experiments. The protocol was approved by the Committee on Animal Research and Ethics of Shanxi University.

## Author Contributions

YL and XQ conceived and designed the experiments. YL and WX performed the experiments and analyzed the data. YL wrote the manuscript.

## Conflict of Interest Statement

The authors declare that the research was conducted in the absence of any commercial or financial relationships that could be construed as a potential conflict of interest.

## Footnotes

1 http://www.hmdb.ca/

2 http://www.kegg.com/

3 http://spin.ccic.ohio-state.edu/index.php/hsqc/

4 http://www.metaboanalyst.ca/

5 http://www.ncbi.nlm.nih.gov/omim

6 http://www.genecards.org/

7 https://david.ncifcrf.gov/
