# Supplementary material for: Deciphering the Mechanical Network of Chronic Atrophic Gastritis: A Urinary Time-Dependent Metabonomics-Based Network Pharmacology Study
Source: Front Physiol. 2019 Aug 6;10:1004. doi: 10.3389/fphys.2019.01004 (PMC6691169; doi:10.3389/fphys.2019.01004)
Supplement: Supplementary file 1 [file Data_Sheet_1.docx]

**Table S1.** Identified metabolites in the rat urine sample based on ^1^H-NMR.

| NO. | Metabolites | Moieties | Chemical shifts |
| --- | --- | --- | --- |
| **1** | Lipids | CH_3_ | 0.87(m) |
| **2** | Isoleucine | CH_3_ | 0.94(t) |
| **3** | Unknown 1 |  | 1.19(s) |
| **4** | 3-Hydroxybutyrate | CH | 1.24(d) |
| **5** | α-Hydroxyisovalerate | CH_3_ | 1.36 (s) |
| **6** | Alanine | βCH_3_ | 1.45 (d) |
| **7** | Acetate | CH_3_ | 1.92(s) |
| **8** | Unknown 7 |  | 1.98(d) |
| **9** | NAG | CH_3_ | 2.04 (s) |
| **10** | Unknown 3 |  | 2.07(s) |
| **11** | Acetone | CH_3_ | 2.23 (s) |
| **12** | Unknown 5 |  | 2.26(s) |
| **13** | Succinate | CH_2_ | 2.41(s) |
| **14** | Citrate | CH_2_,CH_2_ | 2.53 (d), 2.67 (d) |
| **15** | Sarcosine | CH | 2.72(s) |
| **16** | *α*-Ketoglutarate | CH_2_,CH_2_ | 2.44(t),3.02(t) |
| **17** | Unknown 11 |  | 2.83 (s) |
| **18** | Dimethylglycine | N-CH_3_,CH_2_ | 2.93 (s), 3.72 (s) |
| **19** | Creatinine | CH_3,_CH_2_ | 3.05 (s),4.06 (s) |
| **20** | Unknown 4 |  | 3.18(s) |
| **21** | Choline | N(CH_3_)_3_,CH_2_, CH_2_ | 3.23 (s),3.97 (s),3.54 (s) |
| **22** | Taurine | S-CH_2,_N-CH_2_ | 3.26 (t),3.42 (t) |
| **23** | Unknown 9 |  | 3.51(s) |
| **24** | Unknown 10 |  | 3.66(s) |
| **25** | Creatine | CH_2_ | 3.92(s) |
| **26** | Hippurate | CH_2_,CH, CH | 3.98(d),7.56(t),7.85(d) |
| **27** | Unknown 8 |  | 4.02(s) |
| **28** | Lactate | βCH_3_ | 4.13 (d) |
| **29** | Unknown 2 |  | 4.45(s) |
| **30** | N-Methylnicotinamine | CH_3,_CH,CH,CH | 4.48(s),8.9(d),8.97(d),9.29(s) |
| **31** | Unknown 6 |  | 5.26(s) |
| **32** | Allantoin |  | 5.39(s) |

a. s: singlet, d: doublet, t: triplet, q: quartet, m: multiplet, dd: doublet of doublet.

**Table S2**. 57 upstream proteins collected from Metscape based on the related metabolites.

| NO. | Gene | Protein | Metabolites |
| --- | --- | --- | --- |
| 1 | AARS | Alanine--tRNA ligase, cytoplasmic | Alanine |
| 2 | AARS2 | Alanine--tRNA ligase, mitochondrial | Alanine |
| 3 | ACY1 | Aminoacylase-1 | Alanine |
| 4 | AGXT | Serine--pyruvate aminotransferase | Alanine |
| 5 | AGXT2 | Alanine--glyoxylate aminotransferase 2 | ɑ-ketoglutarate |
|  |  |  | Alanine |
| 6 | ALDH5A1 | Succinate-semialdehyde dehydrogenase, mitochondrial | Succinate |
| 7 | ASPH | Aspartyl/asparaginyl beta-hydroxylase | ɑ-ketoglutarate |
|  |  |  | Succinate |
| 8 | BHMT | Betaine--homocysteine S-methyltransferase 1 | Dimethylglycine |
| 9 | BHMT2 | S-methylmethionine--homocysteine S-methyltransferase | Dimethylglycine |
| 10 | CKB | Creatine kinase B-type | Creatine |
| 11 | CKBE | Chlorophyll a-b binding protein E | Creatine |
| 12 | CKM | Creatine kinase M-type  Creatine kinase U-type, mitochondrial | Creatine |
| 13 | CKMT1A |  | Creatine |
| 14 | CKMT1B | Creatine kinase U-type, mitochondrial | Creatine |
| 15 | CKMT2 | Creatine kinase S-type, mitochondrial | Creatine |
| 16 | COMT | Catechol O-methyltransferase | Hippurate |
| 17 | DLD | Dihydrolipoyl dehydrogenase, mitochondrial | ɑ-ketoglutarate |
| 18 | DLST | Dihydrolipoyllysine-residue succinyltransferase component of 2-oxoglutarate dehydrogenase complex, mitochondrial | ɑ-ketoglutarate |
| 19 | DMGDH | Dimethylglycine dehydrogenase, mitochondrial | Dimethylglycine |
| 20 | GAMT | Guanidinoacetate N-methyltransferase | Creatine |
| 21 | GGT1 | Glutathione hydrolase 1 proenzyme | Alanine |
| 22 | GGT2 | Glutathione hydrolase 2 | Alanine |
| 23 | GGT3 | Glutathione hydrolase 3 | Alanine |
| 24 | GGTL3 | **Glutathione hydrolase 7** | Alanine |
| 25 | GGTL4 | Glutathione hydrolase light chain 2 | Alanine |
| 26 | GGTLA1 | Glutathione hydrolase 5 proenzyme | Alanine |
| 27 | GLUD1 | Glutamate dehydrogenase 1, mitochondrial, GDH 1 | ɑ-ketoglutarate |
| 28 | GLUD2 | Glutamate dehydrogenase 2, mitochondrial, GDH 2 | ɑ-ketoglutarate |
| 29 | GLUDP5 | Glutamate dehydrogenase 5, mitochondrial | ɑ-ketoglutarate |
| 30 | GOT1 | Aspartate aminotransferase, cytoplasmic, cAspAT | ɑ-ketoglutarate |
| 31 | GOT2 | Aspartate aminotransferase, mitochondrial, mAspAT | ɑ-ketoglutarate |
| 32 | GPT | Alanine aminotransferase 1, ALT1 | ɑ-ketoglutarate |
|  |  |  | Alanine |
| 33 | GPT2 | Alanine aminotransferase 2, ALT2 | ɑ-ketoglutarate |
|  |  |  | Alanine |
| 34 | HIF1AN | Hypoxia-inducible factor 1-alpha inhibitor | ɑ-ketoglutarate |
|  |  |  | Succinate |
| 35 | IDH1 | Isocitrate dehydrogenase [NADP] cytoplasmic, IDH | ɑ-ketoglutarate |
| 36 | IDH3A | Isocitrate dehydrogenase [NAD] subunit alpha, mitochondrial | ɑ-ketoglutarate |
| 37 | IDH3A | Isocitrate dehydrogenase [NAD] subunit alpha, mitochondrial | ɑ-ketoglutarate |
| 38 | IDH3B | Isocitrate dehydrogenase [NAD] subunit beta, mitochondrial | ɑ-ketoglutarate |
| 39 | IDH3G | Isocitrate dehydrogenase [NAD] subunit gamma, mitochondrial | ɑ-ketoglutarate |
| 40 | KLK1 | Kallikrein 1-related peptidase b1 | Alanine |
| 41 | KLK2 | Tonin | Alanine |
| 42 | L2HGDH | L-2-hydroxyglutarate dehydrogenase, mitochondrial | ɑ-ketoglutarate |
| 43 | NAGS | N-acetylglutamate synthase, mitochondrial | Alanine |
| 44 | OGDH | 2-oxoglutarate dehydrogenase, mitochondrial | ɑ-ketoglutarate |
| 45 | OGDHL | 2-oxoglutarate dehydrogenase-like, mitochondrial | ɑ-ketoglutarate |
| 46 | OXCT1 | Succinyl-CoA:3-ketoacid coenzyme A transferase 1, mitochondrial | Succinate |
| 47 | OXCT2 | Succinyl-CoA:3-ketoacid coenzyme A transferase 2, mitochondrial | Succinate |
| 48 | PDPR | Pyruvate dehydrogenase phosphatase regulatory subunit, mitochondrial | Dimethylglycine |
| 49 | PHYH | Phytanoyl-CoA dioxygenase, peroxisomal | Succinate |
| 50 | SARDH | Sarcosine dehydrogenase, mitochondrial, SarDH | Dimethylglycine |
| 51 | SDHA | Succinate dehydrogenase [ubiquinone] flavoprotein subunit, mitochondrial | Succinate |
| 52 | SDHB | Succinate dehydrogenase [ubiquinone] iron-sulfur subunit, mitochondrial | Succinate |
| 53 | SDHC | Succinate dehydrogenase cytochrome b560 subunit, mitochondrial | Succinate |
| 54 | SDHD | Succinate dehydrogenase [ubiquinone] cytochrome b small subunit, mitochondrial | Succinate |
| 55 | SUCLA2 | Succinate--CoA ligase [ADP-forming] subunit beta, mitochondrial | Succinate |
| 56 | SUCLG1 | Succinate--CoA ligase [ADP/GDP-forming] subunit alpha, mitochondrial | Succinate |
| 57 | SUCLG2 | Succinate--CoA ligase [GDP-forming] subunit beta, mitochondrial | Succinate |

**Table S3**. 148 proteins related to CAG found from OMIM and Genecard databases.

| NO. | Gene | Protein | PDD ID | Source | References |
| --- | --- | --- | --- | --- | --- |
| 1 | IL1B | Interleukin-1 beta | 5MVZ | Genecard | Libyan Journal of Medicine, 2016, 11(1):31576. |
| 2 | ILIRN | Interleukin-1 receptor antagonist protein | 4P0J | Genecard | Libyan Journal of Medicine, 2016, 11(1):31576. |
| 3 | TNF | Tumor necrosis factor | 5M2I | Genecard | World Journal of Gastroenterology, 2004, 10(9):1256. |
| 4 | IL6 | Interleukin-6 | 5FUC | Genecard | Acta gastro-enterologicaBelgica, 2015, 78(2):212-218. |
| 5 | TP53 | Cellular tumor antigen p53 | 4MZR | Genecard | International Journal of Clinical\s&\sexperimental Pathology, 2015, 8(9):10468-10474. |
| 6 | ALB | Serum albumin | 5X52 | Genecard | Peritoneal Dialysis International Journal of the International Society for Peritoneal Dialysis, 2001, 21 Suppl 3(1):S152-156. |
| 7 | IL10 | Interleukin-10 | 5BOW | Genecard | Cancer Epidemiology Biomarkers & Prevention, 2007, 16(12):2631-2636. |
| 8 | IFNG | Interferon gamma | 5MSK | Genecard | European Journal of Cancer Prevention, 2008, 17(2):178-183. |
| 9 | CD40LG | CD40 ligand | 3QD6 | Genecard | Kidney International, 1999, 55(4):1543-1552. |
| 10 | HLA-DRB1 | HLA class II histocompatibility antigen, DRB1-14 beta chain | 4I5B | Genecard | Digestive and Liver Disease, 2010, 42(12):854-859. |
| 11 | CXCL8 | Interleukin-8 | 5WDZ | Genecard | World Journal of Gastroenterology, 2010, 16(14):1788-1794. |
| 12 | AIRE | Autoimmune regulator | 2LRI | Genecard | Molecular Endocrinology, 1998, 12(8):1112-1119. |
| 13 | GAST | Gastrin | 5WRJ | Genecard | Digestive Diseases and Sciences, 2003, 48(1):36-46. |
| 14 | CDH1 | Cadherin-1 | 5I8D | Genecard | Arquivos de Gastroenterologia, 2010, 47(1):7-12. |
| 15 | CHGA | Chromogranin-A | 5DMK | Genecard | Karger, 2001, 64:71–74. |
| 16 | PRTN3 | Myeloblastin | 1FUJ | Genecard | Respiratory Research, 2018, 19(1):180-190. |
| 17 | KRAS | GTPase KRas | 5O2S | Genecard | World Journal of Microbiology and Biotechnology, 2016, 32(6):92-100. |
| 18 | S100A8 | Protein S100-A8 | 5W1F | Genecard | Helicobacter, 2017, 22(5):e12426. |
| 19 | TGFB1 | Transforming growth factor beta-1 | 5V5N | Genecard/OMIM | Beijing Da XueXue Bao, 2009, 41(6):635-639. |
| 20 | PGA3 | Pepsin A-3 | 3UTL | Genecard | Scandinavian Journal of Gastroenterology, 1989, 24(7):870-876. |
| 21 | PGA5 | Pepsin A-5 | 5WF9 | Genecard |  |
| 22 | PGA4 | Pepsin A-4 | 5C9D | Genecard |  |
| 23 | GHRL | Appetite-regulating hormone | 2JSH | Genecard/OMIM | PLOS ONE, 2013, 8. |
| 24 | PTGS2 | Prostaglandin G/H synthase 2 | 5U6X | Genecard | National Medical Journal of China, 2006, 86(38):2683-2689. |
| 25 | HLA-DQB1 | HLA class II histocompatibility antigen, DQ beta 1 chain | 5KSU | Genecard | Journal of Gastroenterology and Hepatology, 2006, 21(2):420-424. |
| 26 | CDX2 | Homeobox protein CDX-2 | 6ES2 | Genecard/OMIM | Digestive Diseases & Sciences, 2011, 56(4):1119-1126. |
| 27 | FOXP3 | Forkhead box protein P3 | 4WK8 | Genecard | Chinese Medical Journal, 2010,123(24):3545-3549. |
| 28 | FAS | Tumor necrosis factor receptor superfamily member 6 | 5TLJ | Genecard/OMIM | BMC Medical Genetics,2011, 12(1):112-112. |
| 29 | PGC | Gastricsin | 1AVF | Genecard/OMIM | PLoS ONE, 2014, 9(12):e115955. |
| 30 | IL2RA | Interleukin-2 receptor subunit alpha | 5M5E | Genecard | Hepato-gastroenterology, 1996, 43(12):1665-1670. |
| 31 | IL4 | Interleukin-4 | 3QB7 | Genecard | Journal of Gastroenterology & Hepatology, 2010, 22(5):729-737. |
| 32 | ADH7 | Alcohol dehydrogenase class 4 mu/sigma chain | 4I5D | Genecard | Alcoholism: Clinical and Experimental Research, 1994,18(4):795-798. |
| 33 | ODC1 | Ornithine decarboxylase | 4ZGZ | Genecard | Cancer Epidemiology Biomarkers & Prevention, 2005, 14(2):451-458. |
| 34 | IL5 | Interleukin-5 | 5T1T | Genecard | Medical Science Monitor International Medical Journal of Experimental & Clinical Research, 2000, 6(6):1128-1135. |
| 35 | CXCL1 | Growth-regulated alpha protein | 5GJE | Genecard | Modern Pathology, 2010, 23(8):1136-1146. |
| 36 | TLR4 | Toll-like receptor 4 | 4G8A | Genecard | Clinics and Research in Hepatology and Gastroenterology, 2014, 38(3):366-371. |
| 37 | CTSW | Cathepsin W | 5MAE | Genecard | World Journal of Gastroenterology, 2005, 11(38):5951-5957. |
| 38 | NOS2 | Nitric oxide synthase, inducible | 5XN3 | Genecard | BMC Gastroenterology, 2010, 10(1):64-72. |
| 39 | LTA | Lymphotoxin-alpha | 4MXV | Genecard | apanese Population. Helicobacter, 2009,14(6)571-579. |
| 40 | IL18 | Interleukin-18 | 3WO3 | Genecard | Microbial Pathogenesis, 2013, 65:7-13. |
| 41 | HLA-B | HLA class I histocompatibility antigen, B-7 alpha chain | 5C09 | Genecard | Infection and Immunity, 1999, 67(6):2969-2974. |
| 42 | ACE | Angiotensin-converting enzyme | 5AM8 | Genecard/OMIM | Asian Pacific Journal of Cancer Prevention, 2005, 6(4):464-467. |
| 43 | IL1A | Interleukin-1 alpha | 5J12 | Genecard | Molecular Medicine Reports, 2012, 5(5):1335-1339. |
| 44 | MUC2 | Mucin-2 | 5VKI | Genecard/OMIM | European Journal of Cancer, 2012, 48(1):0-120. |
| 45 | CTNNA1 | Catenin alpha-1 | 4ONS | Genecard | Journal of Gastroenterology and Hepatology, 2003, 18(5):534-539. |
| 46 | MAP3K6 | Mitogen-activated protein kinase kinasekinase 6 | 5V19 | Genecard | Lansoprazole promotes gastric mucosal cell proliferation and migration by activating p44/p42 mitogen-activated protein kinase, 2004, 12(1):93-99. |
| 47 | NOD2 | Nucleotide-binding oligomerization domain-containing protein 2 | 4UDE | Genecard | Cellular Microbiology, 2010, 8(7):1188-1198. |
| 48 | CAT | Catalase | 5M8L | Genecard | Bratislavskelekarskelisty, 1994, 95(1):9-14. |
| 49 | IL2 | Interleukin-2 | 5UZB | Genecard | Helicobacter, 2005, 10(3):172-178. |
| 50 | MUC5AC | Mucin-5AC | 2ERJ | Genecard | Pathology Research & Practice, 2005, 201(10):665-672. |
| 51 | STAT3 | Signal transducer and activator of transcription 3 | 4ZIA | Genecard/OMIM |  |
| 52 | MIF | Macrophage migration inhibitory factor | 5CG4 | Genecard | The Journal of Infectious Diseases, 2004, 190(2):293-302. |
| 53 | MMP9 | Matrix metalloproteinase-9 | 4WZV | Genecard | Chinese journal of contemporary pediatrics, 2010, 12(4):262-266. |
| 54 | NFKB1 | Nuclear factor NF-kappa-B p105 subunit | 3JV4 | Genecard | Biochimica Et Biophysica Acta, 2018, 1865(4). |
| 55 | MUC1 | Mucin-1 | 2ACM | Genecard | European Journal of Cancer, 2012, 48(1):0-120. |
| 56 | BGLAP | Osteocalcin | 4MZZ | Genecard | Journal of Nutrition Health & Aging, 1998, 2(2):73-78. |
| 57 | HLA-C | HLA class I histocompatibility antigen, Cw-7 alpha chain | 5VGD | Genecard | Arquivos de Gastroenterologia, 2017, 54(4):297-299. |
| 58 | IL17A | Interleukin-17A | 5NAN | Genecard | Journal of Gastrointestinal & Liver Diseases Jgld, 2012, 21(3):243. |
| 59 | MUC6 | Mucin-6 | 2FO4 | Genecard/OMIM | European Journal of Cancer, 2012, 48(1):0-120. |
| 60 | IL15 | Interleukin-15 | 4GS7 | Genecard | Journal of Pathology, 2016, 240(4):425-436. |
| 61 | SOD1 | Superoxide dismutase [Cu-Zn | 4L05 | Genecard | Neoplasma, 1999, 46(2):100-104. |
| 62 | MUC4 | Mucin-4 | 5WZR | Genecard | The Journal of Pathology, 2016. |
| 63 | CYP1A2 | Cytochrome P450 1A2 | 2HI4 | Genecard | Oncology Reports, 2004, 12(6):1335. |
| 64 | ICAM1 | Intercellular adhesion molecule 1 | 5MZA | Genecard/OMIM | Zhonghua Zhong Liu ZaZhi, 2012, 34(3):192-195. |
| 65 | GAD1 | Glutamate decarboxylase 1 | 5EUJ | Genecard |  |
| 66 | HSPD1 | 60 kDa heat shock protein, mitochondrial | 5FWL | Genecard | Yonsei Medical Journal, 2013, 54(6). |
| 67 | CCL5 | C-C motif chemokine 5 | 5L2U | Genecard | American Journal of Pathology, 2011, 178(4):0-1452. |
| 68 | VEGFA | Vascular endothelial growth factor A | 5T89 | Genecard | Journal of Gastroenterology and Hepatology, 2010, 25(4):795-799. |
| 69 | HLA-A | HLA class I histocompatibility antigen, A-2 alpha chain | 5KNM | Genecard | Annals of Internal Medicine, 1983, 99(6). |
| 70 | CASR | Extracellular calcium-sensing receptor | 5XAX | Genecard | Hemodialysis International International Symposium on Home Hemodialysis, 2010, 13(2):176-180. |
| 71 | ADA | Adenosine deaminase | 5J3J | Genecard/OMIM | Anticancer research, 2010, 30(6):2347-2349. |
| 72 | IL2RB | Interleukin-2 receptor subunit beta | 5T5W | Genecard | PLoS Genetics, 2011, 7(8):e1002216. |
| 73 | BCL2 | Apoptosis regulator Bcl-2 | 5TWA | Genecard | Journal of Gastroenterology and Hepatology, 2005, 20(11):1674-1678. |
| 74 | TPH1 | Tryptophan 5-hydroxylase 1 | 4ZEL | Genecard | Transactions of the American Clinical & Climatological Association, 2012, 123:268. |
| 75 | TFF2 | Trefoil factor 2 | 4QO3 | Genecard/OMIM | World Journal of Gastroenterology, 2003, 9(5):910-914. |
| 76 | CCL2 | C-C motif chemokine 2 | 5COR | Genecard | Scientific Reports, 2013, 3:1543-1550. |
| 77 | DDC | Aromatic-L-amino-acid decarboxylase | 3RBL | Genecard | Nippon rinsho. Japanese journal of clinical medicine, 1996, 54(6):1568-1573. |
| 78 | HLA-DQA1 | HLA class II histocompatibility antigen, DQ alpha 1 chain | 4D8P | Genecard | Journal of Digestive Diseases, 2009, 10. |
| 79 | CYP21A2 | Steroid 21-hydroxylase | 5VBU | Genecard | Human Immunology, 2013, 74(6):783-786. |
| 80 | GAD2 | Glutamate decarboxylase 2 | 5V2V | Genecard |  |
| 81 | CYP11A1 | Cholesterol side-chain cleavage enzyme, mitochondrial | 3NA1 | Genecard | Laboratory Investigation, 2015, 95(6):660-671. |
| 82 | SPP1 | Osteopontin | 3CXD | Genecard/OMIM | PLoS ONE, 2014, 9(12):e114005. |
| 83 | TNFSF13B | Tumor necrosis factor ligand superfamily member 13B | 4ZCH | Genecard | The Journal of Immunology, 2014, 193(11):5584-5594. |
| 84 | CYP17A1 | Steroid 17-alpha-hydroxylase/17,20 lyase | 5IRV | Genecard |  |
| 85 | TIMP1 | Metalloproteinase inhibitor 1 | 5UWN | Genecard | Journal of Clinical Otorhinolaryngology, 2005, 19(14):633-635. |
| 86 | IL11 | Interleukin-11 | 1LK3 | Genecard/OMIM | American ournal of Physiology - Gastrointestinal and Liver Physiology, 2018. |
| 87 | MUC3A | Mucin-3A | 2OYP | Genecard | Gut, 2011, 60(11):1607-1608. |
| 88 | MMP2 | 72 kDa type IV collagenase | 4H82 | Genecard | Disease Markers, 2014, 2014:1-9. |
| 89 | CDKN2A | Cyclin-dependent kinase inhibitor 2A | 5VCX | Genecard | World Journal of Gastroenterology, 2002, 8(3):423-425. |
| 90 | NLRP5 | NACHT, LRR and PYD domains-containing protein 5 | 2NAQ | Genecard | The Journal of Immunology, 2002, 169(3):1640-1646. |
| 91 | ITGAL | Integrin alpha-L | 4NEN | Genecard | Tissue Antigens, 2005, 65(3):4. |
| 92 | EGF | Pro-epidermal growth factor | 5EEQ | Genecard | Medical Science Monitor International Medical Journal of Experimental & Clinical Research, 2002, 8(8):CR53-8. |
| 93 | CDKN1B | Cyclin-dependent kinase inhibitor 1B | 5UQ3 | Genecard | Chinese journal of gastrointestinal surgery, 2016, 19(11):1217. |
| 94 | IL21 | Interleukin-21 | 5T7Q | Genecard | Acta gastro-enterologicaBelgica, 2015, 78(2):212-218. |
| 95 | MTHFR | Methylenetetrahydrofolate reductase | 6FNU | Genecard | Cancer Epidemiology Biomarkers & Prevention, 2007, 16(11):2416-2424. |
| 96 | CALCA | Calcitonin gene-related peptide 1 | 5II0 | Genecard | Digestive Diseases & Sciences, 2007, 52(2):411-417. |
| 97 | GSTP1 | Glutathione S-transferase P | 4G9H | Genecard | Cancer epidemiology, biomarkers &prevention : a publication of the American Association for Cancer Research, cosponsored by the American Society of Preventive Oncology, 2005, 14(2):451. |
| 98 | EGFR | Epidermal growth factor receptor | 5KWG | Genecard | International Journal of Experimental Pathology, 2010, 83(5):257-263. |
| 99 | CXCL5 | C-X-C motif chemokine 5 | 2MGS | Genecard | Infection and Immunity, 2001, 69(1):81-88. |
| 100 | HBEGF | Proheparin-binding EGF-like growth factor | 2M8S | Genecard | Medical Science Monitor International Medical Journal of Experimental & Clinical Research, 2002, 8(8):CR53-8. |
| 101 | HSPB1 | Heat shock protein beta-1 | 4X9L | Genecard | Digestive Diseases and Sciences, 2013, 58(2):381-388. |
| 102 | AICDA | Single-stranded DNA cytosine deaminase | 5W0U | Genecard | Gastric Cancer, 2010, 13(1):43-49. |
| 103 | GH1 | Somatotropin | 1HGU | Genecard |  |
| 104 | PPARG | Peroxisome proliferator-activated receptor gamma | 5U5L | Genecard | Hybridoma, 2010, 29(4):341. |
| 105 | IL23A | Interleukin-23 subunit alpha | 5MJ4 | Genecard/OMIM | Chinese Journal of Cellular & Molecular Immunology, 2016, 32(4):523-526. |
| 106 | PCNA | Proliferating cell nuclear antigen | 5T9D | Genecard | Chinese journal of integrated traditional and Western medicine , 2008, 28(3):225. |
| 107 | TGFA | Protransforming growth factor alpha | 5KN5 | Genecard/OMIM | The Korean Journal of Gastroenterology, 2007, 49(49):209-224. |
| 108 | CCL20 | C-C motif chemokine 20 | 5L7M | Genecard | Infection and Immunity, 2007, 75(9):4357-4363. |
| 109 | KRT7 | Keratin, type II cytoskeletal 7 | 4ZRY | Genecard | World Journal of Gastroenterology Wjg, 2006, 12(12):1865. |
| 110 | KRT19 | Keratin, type I cytoskeletal 19 | 3TNU | Genecard | World Journal of Gastroenterology Wjg, 2006, 12(12):1865. |
| 111 | ERCC6 | DNA excision repair protein ERCC-6 | 4CVO | Genecard | Oncotarget, 2017, 8(26):43140-43152. |
| 112 | RUNX3 | Runt-related transcription factor 3 | 5URN | Genecard/OMIM | Cancer, 2012, 118(22). |
| 113 | ANXA1 | Annexin A1 | 2ZOC | Genecard | Mediators of Inflammation, 2013, 2013:1-11. |
| 114 | ALDH2 | Aldehyde dehydrogenase, mitochondrial | 4KWF | Genecard | Cancer Epidemiology Biomarkers & Prevention, 2005, 14(2):451-458. |
| 115 | OGG1 | N-glycosylase/DNA lyase | 5H0K | Genecard/OMIM | International Journal of Epidemiology, 2008, 37(6):1316-1325. |
| 116 | XDH | Xanthine dehydrogenase/oxidase | 5BQP | Genecard |  |
| 117 | XPC | DNA repair protein complementing XP-C cells | 5Y3R | Genecard/OMIM | Oncotarget, 2016, 7(6):6972-6983. |
| 118 | PDX1 | Pancreas/duodenum homeobox protein 1 | 2LY9 | Genecard | Gastroenterology, 2008. |
| 119 | ERCC4 | DNA repair endonuclease XPF | 2AQ0 | Genecard | Gene, 2013, 519(2):335-342. |
| 120 | GHR | Growth hormone receptor | 5HYX | Genecard | World Journal of Gastroenterology, 2004. |
| 121 | F13A1 | Coagulation factor XIII A chain | 4KTY | Genecard |  |
| 122 | DDB2 | DNA damage-binding protein 2 | 5V3O | Genecard | Oncotarget, 2016, 7(6):6972-6983. |
| 123 | AREG | Amphiregulin | 2RNL | Genecard | Medical Science Monitor International Medical Journal of Experimental & Clinical Research, 2002, 8(8):CR53-8. |
| 124 | ERCC2 | TFIIH basal transcription factor complex helicase XPD subunit | 5OF4 | Genecard | International ournal of Epidemiology, 2008, 37(6):1316-1325. |
| 125 | FUT2 | Galactoside 2-alpha-L-fucosyltransferase 2 | 4UNI | Genecard | Biochimie, 2013, 95(5):995-1001. |
| 126 | TFF3 | Trefoil factor 3 | 2KLL | Genecard/OMIM | Gene, 2013, 529(1):104-112. |
| 127 | ABO | Histo-blood group ABO system transferase | 5TJL | Genecard | Gastric Cancer, 2003, 6(1):0008-0016. |
| 128 | ERCC8 | DNA excision repair protein ERCC-8 | 2MUT | Genecard | Oncotarget, 2017, 8(26):43140-43152. |
| 129 | GAB1 | GRB2-associated-binding protein 1 | 5EWZ | Genecard | Molecular Carcinogenesis, 2010, 49(10):869-873. |
| 130 | NKX2-1 | Homeobox protein Nkx-2.1 | 2L9R | Genecard |  |
| 131 | FUT3 | Galactoside 3(4)-L-fucosyltransferase | 5KOR | Genecard | Cancer Letters, 2006, 242(2):0-197. |
| 132 | NDUFS5 | NADH dehydrogenase [ubiquinone iron-sulfur protein 5 | 5GPN | Genecard | Journal of Digestive Diseases, 2010, 10(2):99-106. |
| 133 | SAPCD2 | Suppressor APC domain-containing protein 2 | 3QHE | Genecard |  |
| 134 | PDHX | Pyruvate dehydrogenase protein X component, mitochondrial | 2PNR | Genecard |  |
| 135 | FOXJ1 | Forkhead box protein J1 | 5OCN | Genecard | Cancer Cell International, 2019, 19(1). |
| 136 | BIRC3 | baculoviral IAP repeat containing 3 | 3MOD | Genecard/OMIM | Digestive Diseases and Sciences, 2013, 58(2):381-388. |
| 137 | CEACAM5 | carcinoembryonic antigen related cell adhesion molecule 5 | 2QST | Genecard/OMIM | Journal of Histochemistry & Cytochemistry, 2015:0022155415609098. |
| 138 | NODI | nucleotide binding oligomerization domain containing 1 | 3SIX | Genecard |  |
| 139 | CDX1 | caudal type homeobox 1 | 5LUX | Genecard/OMIM | Gastric Cancer, 2001, 4. |
| 140 | IL1B | interleukin 1 beta | 4X38 | Genecard/OMIM | Anticancer Research, 2013, 33(8):3295-3300. |
| 141 | AKT1 | AKT serine/threonine kinase 1 | 4EKL | Genecard/OMIM | Anticancer Research, 2013, 33(8):3295. |
| 142 | IL1RN | interleukin 1 receptor antagonist | 1IRP | Genecard/OMIM | BMC Medical Genetics,2011, 12(1):112-112. |
| 143 | FASLG | Fas ligand | 2NA6 | Genecard/OMIM | Oncotarget, 2015, 6(22). |
| 144 | PTPN11 | protein tyrosine phosphatase, non-receptor type 11 | 5HDE | Genecard/OMIM | Oncotarget, 2015, 6(22). |
| 145 | IL4R | interleukin 4 receptor | 5E4E | Genecard/OMIM | Cancer Causes and Control, 2006, 17(9):1183-1191. |
| 146 | SHH | sonic hedgehog | 2OAW | Genecard/OMIM | Lab Investig, 2003, 83(12):1829. |
| 147 | NOD1 | Nucleotide-binding oligomerization domain-containing protein 1 | 4E9M | OMIM | Clinical & Experimental Medicine, 2010, 10(2):107-112. |
| 148 | MUC5B | Mucin-5B | None | OMIM | European Journal of Cancer, 2012, 48(1):0-120. |


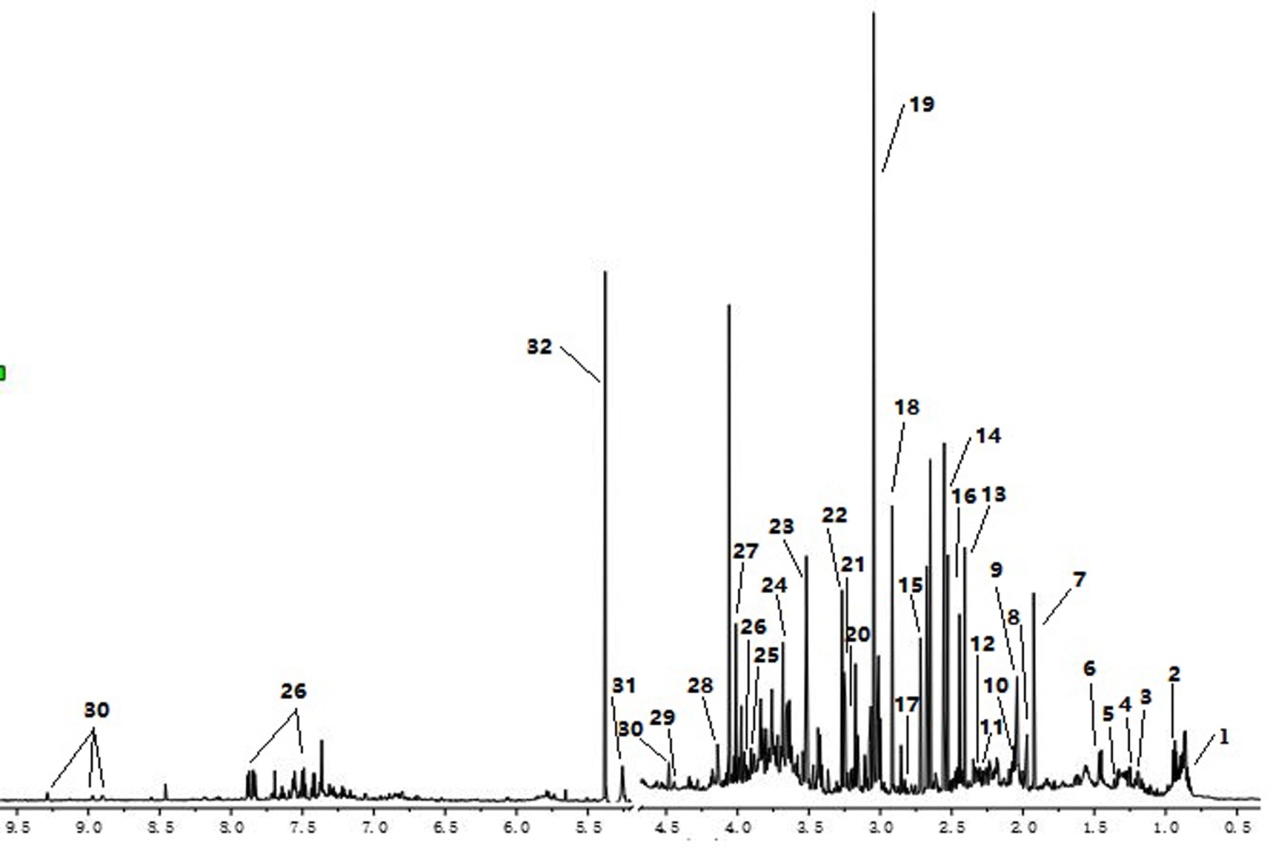


**Figure S1**. Typical ^1^H NMR (600 MHz) spectroscopy of rat urine sample.

**Figure S2**. (A) ^1^H-^1^H COSY spectrum of urine sample, (B) ^1^H-^13^C HSQC spectrum of urine sample.


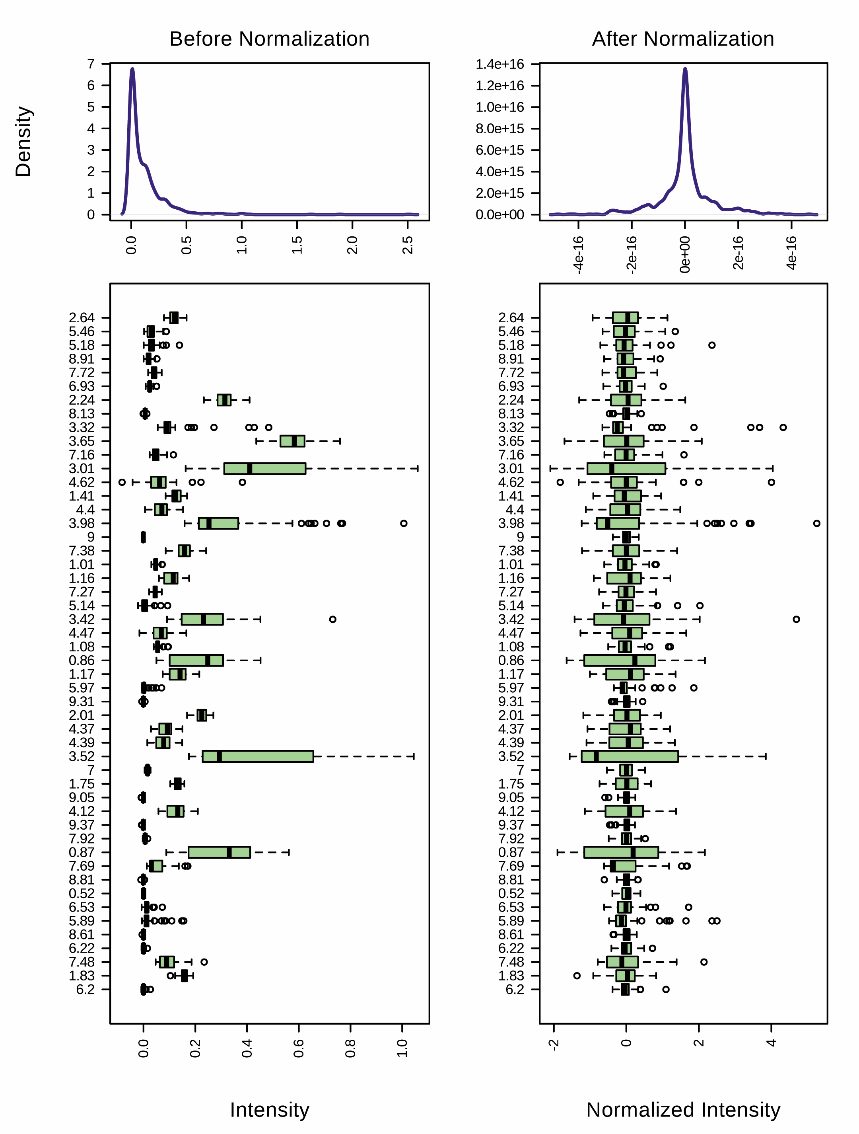


**Figure S3.** The effects before and after normalization.


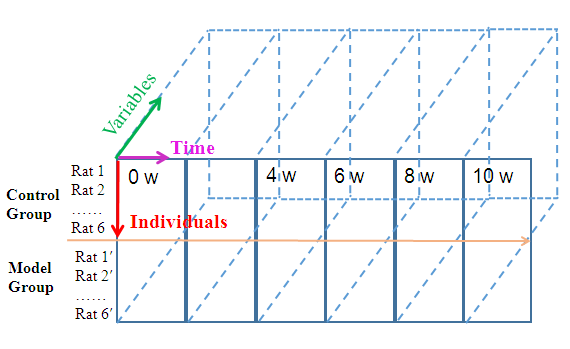


**Figure S**4. The structure of the dataset which is multi-subjects, multi-time points and multi-variables.


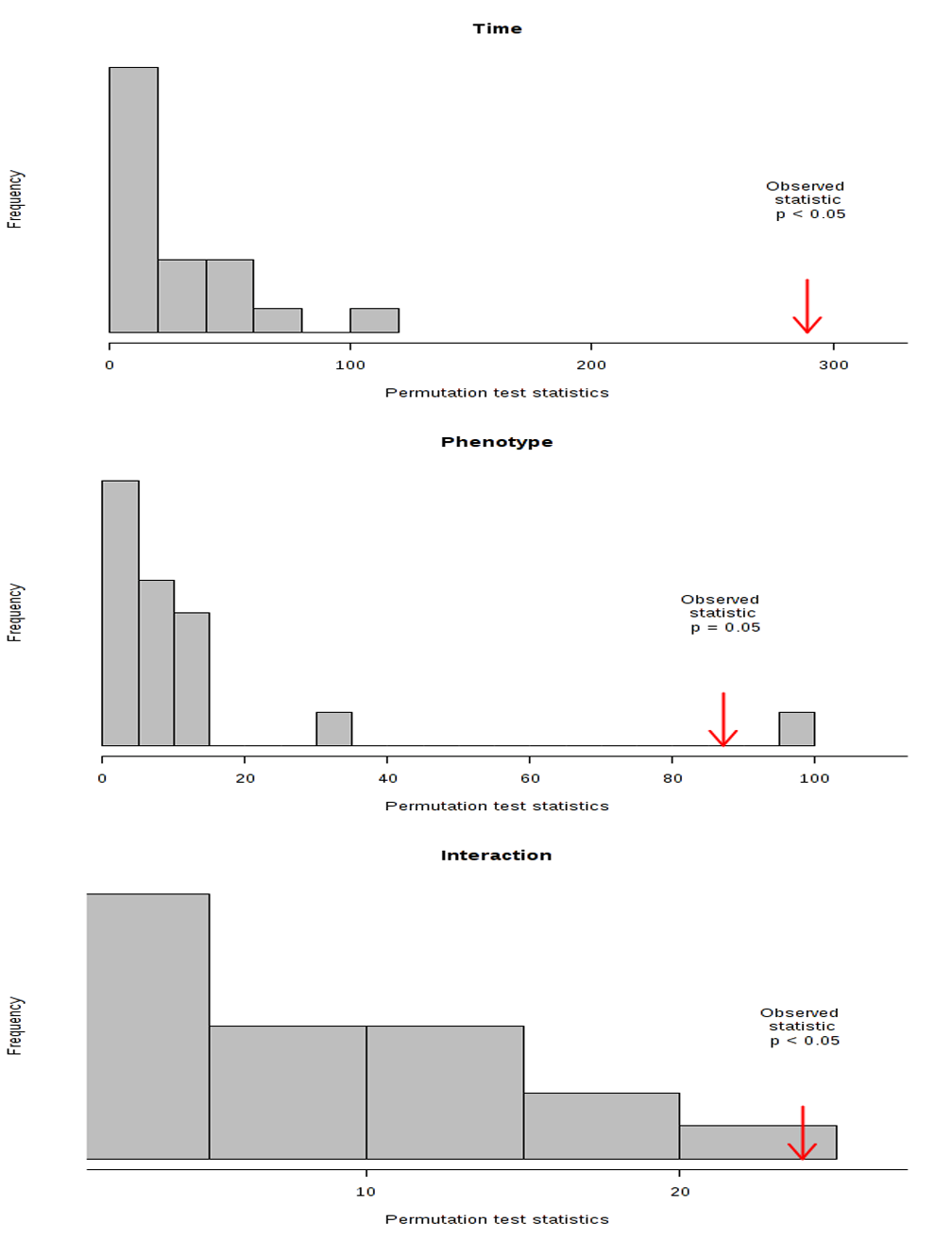


**Figure S5.** Model validations through permutations, as demonstrated by significant levels of *p* < 0.05 for the phenotype, time level, and interaction.


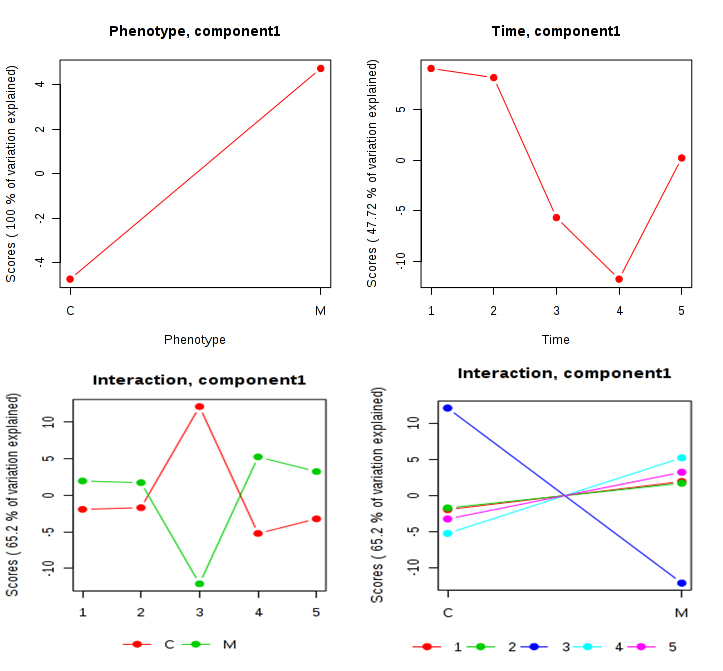


**Figure S6.** ASCA score plots for factor time, factor phenotypes, and their interactions based on PC1 of the corresponding sub models.
